# Bioactive Peptides in Probiotic Fermented Foods: Structure–Activity Relationships and Cardiometabolic Protective Effects

**DOI:** 10.33549/physiolres.935776

**Published:** 2026-08-01

**Authors:** Cheng WANG

**Affiliations:** 1 Morning Stomach Health Technology (Shandong) Co, Ltd, Taian, Shandong, China

**Keywords:** Vascular modulation, Incretin pathways, Lipid regulation, Peptide bioavailability, Fermentation biotechnology

## Abstract

Bioactive peptides generated during probiotic fermentation have emerged as a complex class of food-derived molecules with multifaceted physiological effects relevant to cardiometabolic health. Recent advances in peptidomics, structural analysis, and mechanistic evaluation have revealed that fermentation with lactic acid bacteria, Bacillus species, or mixed cultures produces highly diverse peptide profiles whose activities depend on sequence motifs, hydrophobicity, charge distribution, and resistance to gastrointestinal degradation. These peptides modulate key pathways involved in vascular regulation, glucose metabolism, lipid handling, and inflammatory signaling. Evidence from cellular and animal models consistently demonstrates that selected short sequences inhibit angiotensin converting enzyme, enhance incretin bioactivity through dipeptidyl peptidase IV inhibition, reduce oxidative stress via direct radical scavenging and Nrf2 activation, attenuate inflammatory cascades by neutralizing endotoxin or interfering with immune receptor interactions, and influence lipid metabolism by interacting with bile acids or receptor-regulated transcription factors. Human trials, although heterogeneous in design and outcomes, suggest modest improvements in blood pressure, glucose tolerance, and lipid measures in specific populations, with notable geographical and genetic variability. Controversy remains regarding effect size, reproducibility, and translation of in vitro potency to physiological relevance due to variability in peptide abundance, digestion stability, and differences in habitual diet. Despite such limitations, emerging strategies in controlled fermentation, peptide enrichment, in silico prediction, and combined functional formulations indicate strong potential for targeted development of peptide-rich foods that support cardiometabolic resilience. Continued integration of structural analysis, mechanistic validation, and rigorously designed clinical studies will be essential to clarify their role within food-based preventive strategies.

## Introduction

Probiotic fermented foods including yogurt, cheese, kefir, kimchi, fermented soy products, and cured meats are increasingly valued not only for their extended shelf life and improved flavor but also for their potential health benefits. During fermentation, probiotic microbes such as lactic acid bacteria or Bacillus species break down food proteins into numerous short-chain peptides known as bioactive peptides (BAPs). These peptides, typically 2 to 20 amino acids in length, are embedded within parent proteins and released through microbial proteolysis. Unlike intact dietary proteins, bioactive peptides can interact directly with human physiological pathways and exhibit a range of activities such as antihypertensive, antioxidant, anti-inflammatory, antidiabetic, and lipid-lowering effects. This review critically evaluates the current evidence that bioactive peptides derived from probiotic fermented foods provide cardiometabolic protective benefits, particularly in relation to hypertension, dyslipidemia, glycemic control, and inflammation, and explores the structure–activity relationships that govern these bioactivities.

Scientific interest in peptides from fermented foods has increased markedly over the past two decades. For example, a PubMed search for “bioactive peptides from fermented foods” yields only a small number of studies in the 1990s but reaches hundreds of publications in recent years. This rapid growth in research has revealed a wide range of bioactive sequences produced in diverse fermented food substrates. Dairy products fermented with Lactobacillus or Lactococcus cultures have received particular attention and have yielded well-studied peptides such as the casein-derived tripeptides isoleucine proline proline (IPP) and valine proline proline (VPP). At the same time, many other fermented foods, including fermented meats, fish products, cereals, and legumes, also generate peptides with noteworthy biological activities.

**Fig. 1:**
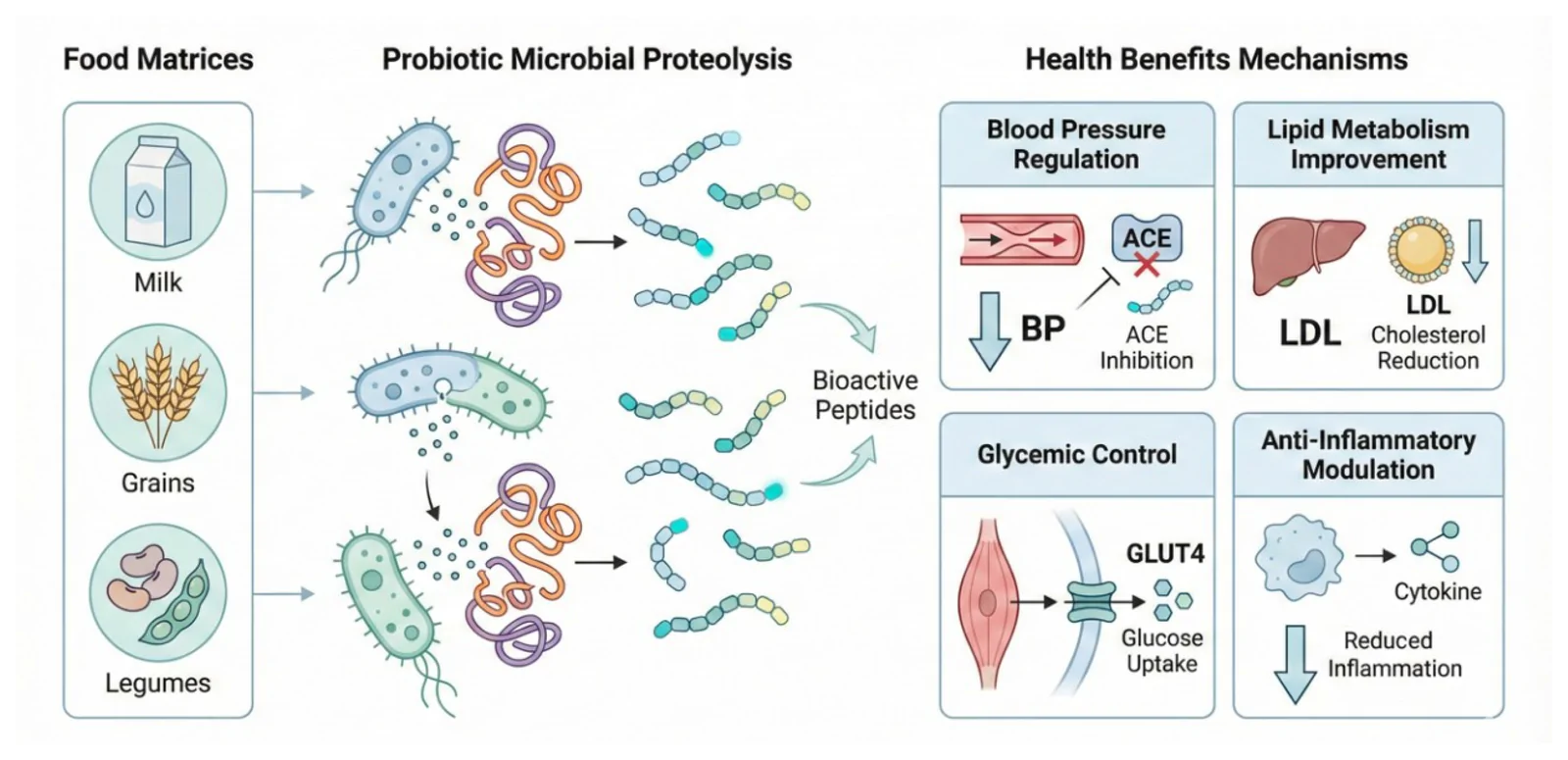
Overview of probiotic fermentation–derived bioactive peptides and their cardiometabolic actions.

A central question is whether intake of these foods or the isolated peptides can meaningfully improve cardiometabolic health in humans. Supporters argue that certain peptide-rich fermented foods function as functional foods that may help regulate blood pressure, glucose metabolism, or lipid handling. Critics point out that clinical evidence remains inconsistent and that the peptide dose achievable in a realistic food serving may be lower than doses used in isolated hydrolysate or supplement studies. Mechanistic uncertainties also persist because peptides must survive digestion, reach relevant biological targets, and exert measurable physiological actions within complex diets and microbiomes. Figure 1 provides an integrated overview of how probiotic fermentation liberates bioactive peptides from diverse food matrices and how these peptides may interact with vascular, metabolic, oxidative, and inflammatory pathways.

In this review, we first survey the major categories of probiotic fermented foods and the bioactive peptides they generate, highlighting specific examples. We then discuss known structure-activity relationships, explaining how peptide sequences and chemical features correlate with biological functions. The core of the review critically evaluates evidence for cardiometabolic benefits of fermented food-derived peptides, organized by outcomes. For each aspect, we analyze supportive studies, null or conflicting evidence, and the degree to which findings from purified peptides, enzymatic hydrolysates, or whole fermented foods can be generalized. We also address challenges in translating peptide bioactivity into functional foods or therapeutics, including production yield, stability, bioavailability, clinical-trial design, and regulatory approval. This narrative review was developed from targeted searches of PubMed, Web of Science, Scopus, Google Scholar, and journal/publisher databases from database inception to May 2026. Search concepts combined terms for fermented foods and microbial cultures (fermented milk, yogurt, kefir, cheese, natto, tempeh, fermented soy, fermented meat, fermented fish, lactic acid bacteria, Bacillus, Lactobacillus, Lactiplantibacillus) with peptide-related terms (bioactive peptide, lactotripeptide, IPP, VPP, ACE-inhibitory peptide, DPP-IV inhibitory peptide, antioxidant peptide, cationic peptide, hydrolysate) and cardiometabolic outcomes (blood pressure, hypertension, glucose, insulin, GLP-1, PYY, lipid, cholesterol, triglyceride, obesity, inflammation, CRP, IL-6, TNF-alpha). Priority was given to human trials, meta-analyses, directly relevant mechanistic studies, and recent reviews. Enzymatic hydrolysate studies were included only when they clarified peptide structure or mechanism; they were not treated as direct evidence for whole fermented foods unless the intervention itself involved a fermented matrix. Studies were excluded when the citation did not match the claim, when the peptide source was unrelated to fermentation, or when the outcome was outside cardiometabolic physiology. Conflicting findings were retained when they materially affected interpretation.

## Bioactive peptides from probiotic fermented foods

Microbial fermentation of protein-rich foods results in the release of a broad range of peptides with measurable biological activities. The profile of bioactive peptides produced is influenced by both the food protein used as the substrate and the specific microbial enzymes that participate in proteolysis. Different categories of fermented foods, including dairy products, meat products, plant-based foods, and marine foods, provide distinct sets of peptides, as summarized in Table 1. This section presents key examples from these major food groups, describing how probiotic fermentation gives rise to peptide formation and highlighting representative bioactivities that have been reported.

Fermented Milk and Dairy Products: Fermented dairy is one of the most extensively studied sources of bioactive peptides. Lactic acid bacteria (LAB) such as Lactobacillus helveticus, Lactococcus lactis, and Streptococcus thermophilus are commonly used to ferment milk into yogurt, kefir, and cheese, where they cleave casein and whey proteins into numerous small peptides. A classic example is the tripeptides IPP (Ile-Pro-Pro) and VPP (Val-Pro-Pro), released from casein by L. helveticus fermentation of milk [1]. These “lactotripeptides” were first identified in fermented milk products and found to exhibit potent angiotensin-converting enzyme (ACE) inhibitory activity in vitro. Subsequent studies demonstrated antihypertensive effects of IPP and VPP in animal models and humans [2–4]. Besides lactotripeptides, fermented dairy yields many other peptides: for instance, fermentation of cheddar cheese by Lactobacillus strains can generate antioxidant sequences like VLPVPQK, which showed strong radical-scavenging capacity in chemical assays and protected cells from oxidative stress. Similarly, yogurt fermented with L. delbrueckii and Streptococcus cultures contains peptides that may contribute to its health effects; peptide profiling of yogurt has revealed fragments with ACE-inhibitory, immunomodulatory, and opioid-like activities [2]. Fermented camel milk and mare’s milk are also under investigation: fermentation of camel milk with Lactiplantibacillus plantarum produced peptides that showed antioxidative and anti-inflammatory properties in vitro. Overall, dairy fermentations produce a rich cocktail of peptides, including antihypertensive “casokinins” from casein and opioid peptides (e.g. β-casomorphins) among others [3]. These peptides are thought to partly underlie the benefits of cultured dairy foods on blood pressure and other cardiometabolic risk factors.

Fermented Meat Products: Traditional fermented meats (such as dry sausages, hams, and fish sauces) are another source of bioactive peptides. The high protein content of meat provides abundant substrate for microbial proteases. For example, Lactobacillus and Staphylococcus species in dry-fermented sausages can hydrolyze muscle proteins into peptides. An illustrative case is a fermented Indonesian beef product (cincalok or Cangkuk beef) inoculated with Bacillus and Lactobacillus, from which two peptides were isolated [4]. Remarkably, oral administration of these peptides to spontaneously hypertensive rats (SHRs) caused a significant blood pressure reduction within hours, with the smaller IVG peptide showing the greatest effect (likely due to easier absorption and ACE-binding) [5]. European dry-cured hams and sausages have also yielded antihypertensive and antioxidant peptides during ripening. For instance, Spanish dry-cured ham contains a peptide AEEEYPDL derived from ham proteins, which was characterized for its antioxidant activity. Another study identified multiple peptides in Italian salami with ACE-inhibitory activity [6]. Fermented fish products share similar potential: in the traditional Asian fermented fish paste bagoong and fermented shrimp paste, researchers have isolated small peptides with ACE-inhibitory and radical-scavenging activities [7]. Likewise, Pekasam, a Malaysian fermented fish, yielded novel hydrophobic peptides with strong DPPH radical scavenging ability [8]. These examples underscore that fermentation by proteolytic bacteria in high-protein foods can release bioactive fragments with antihypertensive, antimicrobial, or antioxidant effects (Table 1). Notably, Bacillus subtilis used in Asian fermentations is highly proteolytic and often generates peptides distinct from those produced by LAB.

**Fig. 2:**
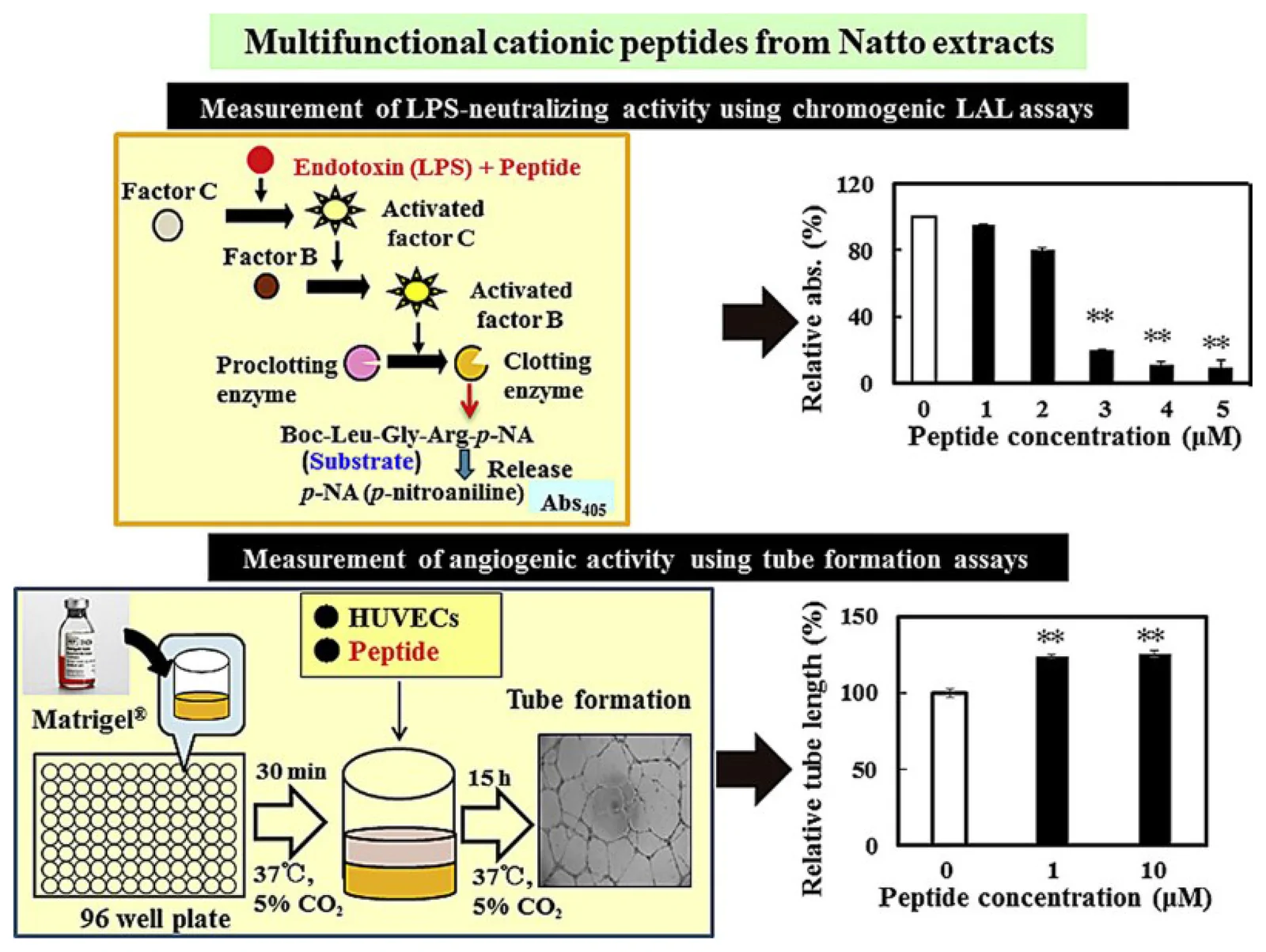
Identification of cationic peptides from traditional Japanese fermented soybean natto extracts. Reproduced from Taniguchi et al. [10] with permission.

Fermented Plant-Based Foods (Legumes and Cereals): Plant proteins in foods such as soybeans, cereals, and legumes can be converted into bioactive peptides through microbial fermentation. Natto, the traditional Japanese fermented soybean food made with Bacillus subtilis, is widely recognized for nattokinase, but recent research has also identified multifunctional cationic peptides in natto extracts [9]. Taniguchi *et al*. isolated six short cationic peptides from natto; sequences such as KFNKYGR and FPFPRPPHQK carry high positive charge due to multiple arginine/lysine residues (Fig. 2) [10]. These peptides neutralized lipopolysaccharide (LPS) and promoted angiogenesis in vitro without cytotoxicity. The finding is mechanistically relevant to metabolic endotoxemia and endothelial repair, but it should be interpreted as preclinical evidence rather than proof that natto intake improves inflammatory biomarkers in humans.

Virtually any protein-rich food that undergoes microbial fermentation can generate bioactive peptides, but the specific outcomes depend on the enzymatic capacities of the microbes and the protein substrates available. Lactic acid bacteria have sophisticated proteolytic systems that systematically break down food proteins into small peptides. L. helveticus, for instance, possesses multiple proteinase genes enabling extensive casein hydrolysis, explaining its efficiency in releasing lactotripeptides and other BAPs in milk. Bacillus species and filamentous fungi have their own proteases that generate different peptide profiles, including longer or more cationic sequences. Table 1 highlights representative bioactive peptides from fermented foods and distinguishes direct fermentation products from peptide fractions or hydrolysates used mainly as mechanistic comparators. This distinction matters because a purified hydrolysate can deliver a higher and more standardized peptide dose than a customary serving of a fermented food.

## Structure–activity relationships of fermented food peptides

Bioactive peptides exert their effects through specific interactions with enzymes, receptors, or radicals, and these interactions are governed by the peptide’s amino acid sequence and structure. A key theme in the literature is that small size and specific amino acid motifs confer high bioactivity [11]. Many food-derived BAPs are short (often 2–9 amino acids), which not only facilitates crossing the intestinal barrier but also allows them to fit into enzyme active sites (in the case of enzyme-inhibitory peptides) [12]. Moreover, certain residues are recurrent in bioactive sequences and seem crucial for activity. This section examines common structure–function trends for peptides with antihypertensive, antioxidant, antidiabetic, or anti-inflammatory actions, highlighting how subtle differences in sequence can influence potency and stability.

ACE-Inhibitory and Antihypertensive Peptides: Perhaps the most studied structure-activity relationships are for ACE-inhibitory peptides (ACEIPs), given the abundance of such peptides in fermented foods [13]. ACE has a catalytic pocket containing a zinc ion and prefers substrates with certain C-terminal residues. Effective ACE-inhibitor peptides often mimic this, typically being dipeptides or tripeptides with hydrophobic/aliphatic or aromatic residues at the C-terminus and a positively charged or hydrophobic N-terminus [14–15]. Proline is frequently found at the C-terminus of potent ACE inhibitors; IPP and VPP both end in Pro-Pro [16]. QSAR studies of di- and tripeptides also support the importance of bulky hydrophobic or aromatic C-terminal residues, including Tyr and Phe, for stronger ACE binding [17]. Peptide length is important: many food-derived ACEIPs are 2–12 amino acids, and tripeptides often balance enzyme binding with intestinal absorption. DGVVYY (Asp-Gly-Val-Val-Tyr-Tyr), identified from Bacillus subtilis-fermented tomato waste proteins, illustrates this pattern because the Val-Val segment and two C-terminal Tyr residues contribute to strong in vitro ACE inhibition [18]. Table 2 summarizes typical motifs in ACE-inhibitory peptides. Notably, ACEIPs often also exhibit stability against digestive enzymes, partly because Pro-containing sequences such as IPP and VPP are relatively resistant to proteolysis [19].

Antioxidant Peptides: Many fermented-food peptides can neutralize free radicals or chelate pro-oxidant metal ions, thereby acting as antioxidants [9]. The structure-activity relationship for antioxidant peptides often revolves around the presence of certain amino acids that can donate electrons or stabilize radical species. Hydrophobic amino acids (like Val, Leu, Ala, Pro) enhance lipid-solubility of the peptide, helping it interact with and protect cell membranes from lipid peroxidation [20]. This partly explains why many antioxidant peptides are rich in hydrophobic residues. Additionally, aromatic residues such as Tyr and Trp can delocalize unpaired electrons across their rings, thus stabilizing radicals. Tyrosine’s phenolic hydroxyl can donate a hydrogen atom to quench radicals, forming a stable tyrosyl radical. Indeed, the presence of Tyr or Trp correlates with high radical scavenging in many peptide studies [21]. For example, the peptide SNLCRPCG (Ser-Asn-Leu-Cys-Arg-Pro-Cys-Gly) derived from fermented chicken feathers exhibited strong antioxidant power largely due to two cysteine residues (Cys) providing thiol (-SH) groups and hydrophobic Leu/Pro facilitating membrane interaction [22]. Cysteine and methionine residues are highly beneficial: they can chelate transition metals and directly quench radicals via their sulfur atoms. Cysteine-containing peptides are known to be potent antioxidants because the thiol group is easily oxidized, thus protecting other targets [23]. The peptide SNLCRPCG’s strong DPPH scavenging was attributed to its thiol groups and hydrophobic character [23]. Histidine (His) is another important residue. Many antioxidant peptides from fermented foods contain His, which can scavenge per se and also enhance binding to oxidized radicals or metals [24]. For instance, an antioxidant peptide from whey fermented by Lactobacillus had the sequence VKEAMAPK; it contains a His analog (Lys) and Glu which could chelate metals, and it was shown to activate Nrf2 by interfering with Keap1 [25]. In summary, antioxidant peptide potency is boosted by (i) hydrophobic/aromatic residues that enhance radical interaction, (ii) cysteine, methionine, or histidine that directly quench radicals or bind metals, and (iii) overall structure that can penetrate to sites of radical generation (e.g. membranes or cytosol). Some fermented food peptides also induce endogenous antioxidant pathways: as mentioned, peptides LY-4, LP-5, VL-9 from fermented bean paste activated the Nrf2 pathway in cells [26]. Such peptides likely possess structural features enabling them to disrupt the Keap1-Nrf2 complex (perhaps by binding Keap1’s pockets via specific amino acid side chains [27]. For example, some peptides with sequences similar to ETKEV can inhibit Keap1, because they present an acidic residue and hydrophobic motif that anchors into Keap1 as demonstrated by in silico docking [28]. This illustrates that beyond direct radical scavenging, peptide structure can allow protein-protein interactions to modulate signaling pathways.

Antidiabetic (DPP-IV Inhibitory) Peptides: A major mechanism for anti-diabetic peptides is inhibition of dipeptidyl peptidase IV (DPP-IV), the enzyme that degrades incretin hormones like GLP-1. DPP-IV cleaves dipeptides from the N-terminus of peptides/proteins, especially when the penultimate position is proline or alanine. Effective DPP-IV inhibitory peptides often resemble the N-terminus of DPP-IV’s substrates or bind to its active site via complementary interactions. Structure-activity studies on DPP-IV inhibitors from food proteins have found that short peptides (2–5 amino acids) with proline or hydroxyproline near the N-terminus tend to be good inhibitors [29]. This is logical, as DPP-IV has a “post-proline” cleaving specificity; peptides containing a Pro at P1 position can sit in the enzyme’s active site and act as competitive inhibitors. For example, the peptide IP (Ile-Pro) is itself a DPP-IV substrate analog and indeed shows inhibitory activity; adding a third residue can improve binding. Many known DPP-IV inhibitory sequences are tripeptides or tetrapeptides with Pro or Ala in the second position and a bulky or hydrophobic first residue (at N-terminus) like Trp, Tyr, or Phe. Trp-Val (WV) and Trp-Leu (WL) are simple dipeptides reported to have notable DPP-IV inhibition [30]. Additionally, positively charged residues can enhance binding to the relatively negative regions of DPP-IV’s active site: some potent inhibitors include Arg or Lys at the C-terminus, which may form salt bridges with the enzyme. In the fermented fish study, among 125 peptides identified, the ones with lowest docking energy to DPP-IV all had favorable structural features: e.g. peptide EPAEAVGDWR (10-mer) included both a hydrophobic segment and a C-terminal Arg, enabling multiple hydrogen bonds and salt bridges in docking. Shorter peptides from that study like IPH (Ile-Pro-His) also showed inhibition (IC_50_ in low mM range), where His provides an imidazole that may stack in the active site and participate in metal coordination if any. Importantly, DPP-IV inhibitors need to survive gut transit to reach the systemic circulation or at least act locally in the intestinal lumen to protect GLP-1. Many DPP-IV inhibitory peptides are relatively short and proline-containing, which (as with ACEIPs) tends to make them more resistant to further proteolysis. Some milk-derived DPP-IV inhibitory peptides also stimulate insulin secretion directly, which suggests structural mimicry of incretin hormones or ability to activate receptors on pancreatic β-cells [31]. While the precise structures for insulinotropic action are not fully clear, it has been observed that certain β-casomorphin-like peptides can bind opioid receptors in the gut, leading to enhanced glucose-dependent insulin release – in these cases, the tyrosine at N-terminus is critical for receptor binding [32]. Thus, structure–activity relationships for antidiabetic peptides involve two angles: enzyme inhibition and hormonal modulation. Table 2 outlines some known structural preferences for these activities. In short, fermented food peptides that help glycemic control often share features with drug-like peptidomimetics: they have the right shape to occupy DPP-IV’s active site or to trigger receptors, and these features usually stem from the presence of strategically placed amino acids [33].

Anti-Inflammatory and Other Bioactivities: Peptides that modulate inflammation or immune responses are another important category, but their evidence base is more mechanistic than clinical. Cationic fermented-food peptides carry multiple basic residues that can bind negatively charged molecules such as LPS or interact with immune-cell surfaces [34]. The natto-derived peptide GEIPRPRPRPQHPE, for instance, has several arginine/lysine residues and can electrostatically neutralize LPS, thereby limiting LPS-driven Toll-like receptor signaling in vitro [35]. The broader antimicrobial-peptide literature supports this charge-dependent LPS-binding mechanism [36]. Pro-Gly-Pro-containing peptides represent a different immunomodulatory pattern: Pro and Gly can create turn structures that interact with chemokine or cytokine-related pathways, but the clinical relevance of such motifs in fermented foods remains uncertain [37–38]. These structural themes justify clinical investigation of inflammatory markers, yet they do not by themselves establish that fermented foods or isolated peptides lower CRP, IL-6, or TNF-alpha in humans.

In summary, although bioactive peptides vary widely, certain structural themes recur: C-terminal Pro or Tyr for ACE inhibition, N-terminal Trp/Tyr or X-Pro motifs for DPP-IV inhibition, positively charged motifs for LPS neutralization, and the inclusion of residues like Cys, His, or Trp for antioxidant effects (Table 2). These patterns reflect the molecular requirements to engage specific biological targets. It also underscores the possibility to optimize peptides or design analogues – for example, enhancing a peptide’s activity by adding a tyrosine at the C-terminus or by cyclizing it to increase stability [39]. The structure–activity understanding is therefore not only academically interesting but also valuable for bioengineering: fermentation conditions or enzyme treatments can be adjusted to enrich for peptides with desired motifs [40]. In the next section, we will connect these structure-informed activities to actual evidence of cardiometabolic protection, examining to what extent these peptides improve health markers and outcomes in experimental and clinical settings.

## Cardiometabolic protective effects of fermented food-derived peptides

A central question is whether the bioactive peptides discussed above measurably improve cardiometabolic health when fermented foods or isolated peptides are consumed. Cardiometabolic health encompasses blood pressure regulation, glucose and insulin homeostasis, lipid profile, body weight/adiposity, and systemic inflammation – all risk factors for cardiovascular disease (CVD) and type 2 diabetes. Here we critically review evidence for peptides’ effects in each of these domains. We integrate results from cell and animal models with findings from human trials and epidemiological studies on fermented foods. A pattern emerges: some fermented food-derived peptides do exert beneficial effects on cardiometabolic markers, particularly blood pressure and vascular function, but the magnitude and consistency of these effects in humans remain a point of debate. We examine both the supportive evidence and the counter-evidence or limitations for each purported benefit. Throughout, we highlight specific examples and the key arguments on each side of the controversy.

### Antihypertensive effects (blood pressure reduction)

One of the most touted benefits of fermented food peptides is blood pressure (BP) reduction via inhibition of the renin-angiotensin system, especially through ACE inhibition. The case for antihypertensive effects initially rested on robust animal data and small human trials: Spontaneously hypertensive rats (SHR), a model for human hypertension, show significant BP decreases when given fermented milk or purified peptides like IPP and VPP [41]. For example, oral administration of IPP to SHR (doses ~3 mg/kg) acutely lowered systolic BP by ~30 mmHg in some studies [42]. These peptides act akin to ACE-inhibitor drugs (though weaker) by blocking the formation of vasoconstrictor angiotensin II and possibly enhancing vasodilatory bradykinin. Animal studies also show that longer-term feeding of peptide-enriched fermented milk can attenuate the development of hypertension. Beyond rodents, small clinical trials in the 1990s and early 2000s reported that fermented milk products containing IPP and VPP produced notable BP reductions in humans. A frequently cited trial in Japan by Hata *et al*. [43] found that hypertensive subjects who drank sour milk containing ~7 mg/day of IPP+VPP experienced an average drop of ~10–14 mmHg in systolic BP after 8 weeks, compared to minimal changes with placebo milk. Another Japanese trial in mild hypertensives observed a ~6 mmHg greater reduction in systolic BP with IPP/VPP-fermented milk vs. control. These early successes, mostly in Japanese populations – led to commercial products and health claims in some countries. A 2015 meta-analysis focusing on Japanese trials (18 studies, n=1194) concluded that IPP/VPP significantly reduce office systolic BP by about −5.6 mmHg on average, with larger effects (−8.4 mmHg) in hypertensive subjects [44]. This meta-analysis found consistency among Japanese studies and no publication bias, strengthening the evidence. It also noted efficacy at relatively low doses (~3–5 mg/day of peptides) commonly achievable in functional foods.

However, as more studies accumulated, especially outside Japan, the picture became more complicated. Several European trials failed to replicate significant blood-pressure lowering with the same peptides. Engberink *et al*. reported no clinically relevant reduction in 24-h ambulatory blood pressure after IPP/VPP supplementation in mildly hypertensive Dutch men [45]. Other controlled European studies similarly reported smaller or null effects, which led EFSA to conclude that a cause-effect relationship between IPP/VPP and maintenance of normal blood pressure had not been established [46]. Later meta-analyses reconciled the divergent literature by showing statistically significant but small mean effects. Qin *et al*. estimated pooled reductions of 1.66 mmHg for systolic blood pressure and 0.76 mmHg for diastolic blood pressure, with 24-h ambulatory systolic blood pressure reduced by about 1.30 mmHg [47]. Cicero *et al*. found that effects were more evident in Asian than European trial populations [44]. These findings suggest that ethnicity/geography, baseline blood pressure, study duration, food matrix, dose, and measurement method all affect the apparent response.

Beyond IPP/VPP, other peptides and fermented foods have shown antihypertensive potential. Fermented sour milk products with different strains (e.g. Lactobacillus plantarum) have yielded peptides that in pilot trials lower BP. A recent Chinese RCT reported that a fermented milk with Lactobacillus plantarum strains providing peptides lowered systolic BP by ~7 mmHg vs placebo over 8 weeks in hypertensive patients [48]. Fermented goat milk tablets rich in 10–15 kDa peptide fractions significantly reduced SBP (−12.5 %) and DBP (−9.3 %) in prehypertensive adults in a trial. These findings suggest peptides beyond just IPP/VPP (perhaps longer peptides or different sequences) can also be effective. In animal models, peptides like the tripeptide IVG from fermented beef mentioned earlier demonstrated acute BP reduction comparable to ACE-inhibitor drugs [4]. Another peptide, RVY (Arg-Val-Tyr) from fermented fish, showed antihypertensive effects in rats by dual ACE and renin inhibition. These mechanistic studies broaden the pool of antihypertensive peptides.

Despite promising examples, controversy persists regarding real-world impact. A mean reduction of approximately 1–2 mmHg is small for an individual and should not be presented as an alternative to antihypertensive treatment. At the population level, however, even modest downward shifts in systolic blood pressure can contribute to cardiovascular-risk reduction when achieved safely across large groups [49]. Larger decreases of about 5 mmHg, reported in selected positive trials or more responsive subgroups, would be clinically more meaningful, but those effects have not been reproduced consistently across regions and trial designs. Correlational dairy-consumption data cannot resolve this uncertainty because fermented dairy also provides calcium, potassium, probiotics, and other nutrients that may influence blood pressure independently of peptides [50–51].

In summary, the controversy lies not in whether lactotripeptides can inhibit ACE, but in whether the effect is large and reproducible enough to support a health claim in diverse human populations. Table 3 summarizes selected antihypertensive evidence, including positive early trials, null European trials, the EFSA assessment, and meta-analytic estimates. The most defensible conclusion is that fermented-food peptides may contribute modestly to blood-pressure management in some contexts, particularly as an adjunct to dietary patterns for individuals with elevated blood pressure, but claims should specify dose, matrix, population, and expected effect size.

### Effects on glycemic control and diabetes

Another promising area is the use of fermented food peptides to improve glycemic control and potentially aid management or prevention of type 2 diabetes. Several mechanisms have been proposed: inhibition of DPP-IV to prolong incretin hormones (GLP-1 and GIP), stimulation of insulin secretion, reduction of inflammatory insulin resistance, and modulation of gut microbiota and gut hormones. The evidence must be separated by source: fermented foods contain peptides together with microbes, organic acids, minerals, and food-matrix components, whereas enzymatic hydrolysates and purified peptide supplements test a more concentrated peptide fraction.

*In vitro* and *ex vivo* evidence strongly supports that fermented-food peptides can inhibit DPP-IV, the enzyme that degrades GLP-1. Multiple studies have fermented milk or other protein sources and then tested the fermentation supernatants for DPP-IV inhibition. For example, Lactobacillus helveticus–fermented milk was shown to have higher DPP-IV inhibitory activity than unfermented milk, indicating generation of inhibitory peptides during fermentation. Specific peptides identified include fragments of casein and whey (e.g. Ile-Pro-Ala, Val-Ala-Pro-Phe, etc.), with IC_50_ values in the low to mid micromolar range, meaning they can substantially inhibit DPP-IV at concentrations potentially achievable in the gut after a serving of fermented dairy. A recent study by Wang *et al*. (2022) on fermented fish (Chouguiyu) explicitly identified 4 novel DPP-IV inhibitory peptides *via* peptidomic analysis, with the most potent (EPAEAVGDWR) having an IC_50_ ~0.1 mM. These peptides interacted with DPP-IV’s active site through multiple bonds (predicted by docking), confirming a direct enzymatic inhibition mode. Additionally, milk fermented with certain probiotic combinations (LAB + yeast) has been found not only to contain DPP-IV inhibitors but also to preserve more GLP-1 during *ex vivo* digestion assays than non-fermented milk. This suggests that fermented products might allow more incretins to survive in the gut to stimulate insulin.

Animal studies provide proof-of-concept that these inhibitory peptides translate into better glucose tolerance. For instance, Power *et al*. [52] reported that oral administration of a whey protein hydrolysate rich in DPP-IV inhibitory peptides to diabetic rats led to improved oral glucose tolerance comparable to a low dose of sitagliptin. Similarly, rodents given fermented milk or fermented camel milk had better fasting glucose and insulin levels than those given non-fermented equivalents. One study on diabetic rats showed that fermented camel milk feeding resulted in significantly reduced blood glucose (~35 % reduction) and HbA1c, along with improved insulin levels, whereas non-fermented camel milk had lesser effects [53]. The authors attributed this to bioactive peptides plus probiotic metabolites in fermented milk enhancing insulin sensitivity and preserving beta-cell function. In the same vein, a recent trial in rats comparing fermented vs non-fermented milk found that only the fermented one upregulated insulin signaling proteins in muscle and liver, likely through both SCFAs and peptides from fermentation. These mechanistic clues indicate peptides may act beyond incretin preservation.

When it comes to human evidence, it is more limited but hints at benefits. Unlike blood pressure, there have been fewer direct peptide trials for glycemic endpoints. However, some indirect evidence is available from fermented dairy consumption studies. Epidemiological data show yogurt consumers have a lower risk of developing type 2 diabetes For instance, a large meta-analysis found that each daily serving of yogurt was associated with an 18 % reduced risk of T2D, even after adjusting for confounders. While yogurt’s benefits could come from many factors, bioactive peptides are one hypothesized factor in yogurt’s positive metabolic profile. Intervention studies in humans have tested fermented dairy products on metabolic syndrome parameters. One study gave prediabetic subjects a fermented milk high in bioactive peptides and GABA for 12 weeks and observed improved insulin resistance (HOMA-IR) and lower fasting blood glucose compared to baseline. Though it lacked a non-fermented control, it indicated fermented milk could beneficially modulate glycemic control. A placebo-controlled trial in Iran with synbiotic yogurt in type 2 diabetics found that after 8 weeks, the synbiotic yogurt group had significantly reduced fasting glucose (~−22 mg/dL) and HbA1c, and modestly improved insulin sensitivity, relative to plain yogurt [54]. While the added fiber/probiotic complicates attribution, it underscores that fermented dairy can be formulated to improve diabetic outcomes.

Focusing on peptides specifically, human evidence is indirect and still limited. Food-protein hydrolysates rich in DPP-IV-inhibitory peptides provide a useful proof-of-mechanism model, but they should not be equated with a usual serving of fermented dairy or soy [52]. Some acute studies of protein or fermented-dairy preloads have reported changes in postprandial glucose, insulin, or incretin responses, yet the active contribution of fermentation-derived peptides is difficult to isolate from intact proteins, amino acids, organic acids, and microbial metabolites. Therefore, DPP-IV inhibition by fermented-food peptides remains a plausible mechanism that needs better human biomarker studies rather than a settled clinical claim.

Insulinotropic and metabolic effects of peptides also extend to weight and fat metabolism indirectly. Some peptides derived from fermentation have shown to activate cellular pathways like AMPK or PPAR that enhance insulin sensitivity and fat oxidation. For example, fermented soybean peptides in cell culture suppressed adipocyte differentiation by downregulating SREBP-1c and FAS gene expression. This could mean less ectopic fat and better insulin sensitivity *in vivo*. And indeed, the doenjang study where overweight individuals consumed fermented soy paste noted, besides antioxidant effects, a trend toward reduced body fat and improved metabolic markers in those consuming it versus placebo. Notably, participants with a certain PPARγ gene variant responded most.

Despite these positive signals, the evidence in humans is still emerging and not uniformly conclusive. Many trials use whole foods such as yogurt that contain not only peptides but also probiotics, minerals, and fermentation metabolites. In a randomized trial, an IPP-rich milk-protein hydrolysate lowered blood pressure in subjects with stage 1 hypertension, but glycemic endpoints were not the primary evidence base for that intervention [55]. This supports the need to avoid transferring conclusions from blood-pressure trials or hydrolysate studies directly to diabetes prevention. If peptides help glycemia, their effect may require a specific metabolic state, a sufficient dose, or synergy with other fermented-food components.

Nevertheless, mechanistic reasoning and animal studies support continued investigation of fermented food peptides as adjuncts for metabolic control. By extending incretin action, DPP-IV-inhibitory peptides resemble a validated drug mechanism, although food-derived peptides are less potent and shorter-lived than pharmaceutical inhibitors. A practical research direction is to test peptide-enriched fermented products with meals while measuring active GLP-1, DPP-IV activity, postprandial glucose, insulin, appetite hormones, and candidate peptide exposure in parallel [56].

In conclusion, supporting evidence indicates that fermented food peptides can improve glycemic indices through DPP-IV inhibition and possibly direct insulinotropic effects, but the human evidence is less mature than the mechanistic literature. Opposing evidence or limitations include inconsistent chronic trial results, uncertainty about peptide survival and absorbed dose, and difficulty separating peptide effects from the broader fermented-food matrix. More human trials in prediabetes or early diabetes should measure peptide exposure, active GLP-1, DPP-IV activity, insulin sensitivity, and inflammatory biomarkers together, so that mechanism and clinical outcome can be linked more directly.

### Clinical evidence on inflammatory biomarkers

Low-grade inflammation is central to cardiometabolic disease because adipose tissue macrophages, hepatocytes, endothelial cells, and immune cells produce cytokines that impair insulin signaling and vascular function [57]. In this context, fermented-food peptides with antioxidant, LPS-neutralizing, or immunomodulatory properties are biologically plausible contributors to lower inflammatory tone. However, human evidence remains much thinner than the in vitro and animal literature. Trials usually test whole fermented products rather than isolated peptides, and inflammatory outcomes are often secondary endpoints.

Among available human studies, fermented camel milk in adolescents with metabolic syndrome improved glucose and insulin-resistance indices and included inflammatory biomarkers such as hs-CRP and TNF-alpha [53]. A recent randomized trial of synbiotic yogurt in adults with metabolic syndrome also evaluated metabolic-syndrome components in a fermented-dairy matrix, but attribution to peptides is complicated by the synbiotic formulation and other dairy constituents [54]. These studies are useful because they move beyond cell models, yet they do not establish a clean peptide-specific anti-inflammatory effect. Evidence for IL-6 is similarly limited and heterogeneous, and null or modest findings should be expected when baseline inflammation is low or interventions are short.

The clinical interpretation is therefore cautious. Fermented-food peptides may contribute to lower CRP, IL-6, or TNF-alpha indirectly by reducing endotoxin signaling, oxidative stress, adipose inflammation, or postprandial metabolic stress, but current human studies are not sufficient to rank anti-inflammatory activity alongside blood-pressure evidence. Future trials should prespecify inflammatory biomarkers, compare fermented with non-fermented controls matched for protein and energy, and quantify candidate peptides in the food and, when possible, in plasma or urine. Recent Physiological Research studies on metabolic syndrome and adipose inflammation reinforce the importance of antioxidant and anti-inflammatory pathways in cardiometabolic physiology [58–59].

### Effects on blood lipids and obesity

Cardiometabolic syndrome involves dyslipidemia and often obesity. Here, the evidence for peptides is less abundant than for blood pressure, but it is relevant because lipid accumulation, oxidative stress, and inflammatory signaling reinforce each other in metabolic syndrome [58–59]. Some fermented-food peptides have been reported to lower cholesterol or triglycerides in experimental systems, either by binding bile acids in the gut or by modulating lipid metabolism genes in the liver. Additionally, antioxidant and anti-inflammatory peptide effects may indirectly improve lipid handling.

Cholesterol-lowering: Human evidence is suggestive but not definitive. Some fermented-dairy interventions report modest changes in LDL cholesterol or triglycerides, but co-interventions such as plant sterols, probiotics, or broader dietary change make peptide attribution difficult. A lipid change of 5–10 % may be meaningful when sustained, yet it should be interpreted in the context of baseline risk and the much larger evidence base for established lipid-lowering therapies [60]. For fermented foods, the stronger conclusion is that peptide-rich matrices may support lipid metabolism as part of a dietary pattern, while isolated peptide efficacy for dyslipidemia remains underdeveloped.

From mechanistic perspectives, peptides may lower cholesterol through: (1) binding bile acids in the gut, promoting their excretion – some plant protein peptides are known to precipitate bile acids, forcing the liver to use more cholesterol to synthesize new bile acids, thus lowering blood LDL. For example, the peptide YPWP from rice bran fermentation was reported to bind taurocholate in vitro, hinting at a bile-binding capacity. (2) Inhibiting cholesterol synthesis enzymes: peptides with structural similarity to HMG-CoA could in theory compete for HMG-CoA reductase. While not as potent as statins, some tripeptides have shown weak HMG-CoA reductase inhibition in test tube assays. (3) Activating hepatic receptors (PPARα, LXR) that upregulate cholesterol clearance – as noted, peptides that activate PPARα would increase ApoA1 and HDL, and reduce TG. One study in obese mice fed fermented milk observed increased PPARα expression in the liver alongside improved lipid profiles, potentially attributable to peptides in the fermented milk interacting with PPAR signaling. (4) Reducing oxidative stress in the liver: oxidative stress can oxidize LDL and reduce HDL functionality; antioxidant peptides might preserve HDL’s ability to mediate cholesterol efflux. It was shown that after consuming fermented soy, overweight individuals had enhanced HDL function and higher antioxidant enzyme levels. This suggests that fermentation products could improve the quality, not just quantity, of cholesterol carriers.

Weight and adiposity: Fermented foods are sometimes associated with anti-obesity effects, but peptide-specific evidence remains preliminary. Peptides could influence adiposity through bile-acid binding, AMPK/PPAR signaling, reduced adipose inflammation, or altered gut-hormone responses. Because adipose tissue macrophages are strongly linked to insulin resistance and metabolic inflammation [57], anti-inflammatory peptide actions are mechanistically attractive. Nevertheless, many animal and cell studies use purified peptides or concentrated extracts that do not match the peptide dose delivered by ordinary fermented foods.

On the satiety front, DPP-IV inhibition could theoretically prolong active GLP-1 and peptide YY signaling, which might reduce appetite or subsequent energy intake. This remains a hypothesis for fermentation-derived peptides rather than an established clinical outcome. Short-term studies of yogurt or fermented dairy may show altered appetite or postprandial responses, but those effects can arise from protein load, viscosity, acidity, probiotics, or matrix structure as well as peptides. Therefore, the manuscript treats appetite and body-weight effects as promising but not yet proven in humans.

In terms of controversy for lipids and weight, it is not as heated mainly because the research is early. But some skepticism is warranted: the lipid changes observed with peptides are relatively small (LDL changes ~5–10 %, TG ~10 %) – such magnitude might or might not significantly reduce CVD risk by itself. Critics might say these changes could be due to other components in fermented foods. A published critical view noted that in many dairy trials, calcium and vitamin D also contribute to cholesterol-lowering and blood pressure effects, so parsing peptide effect is tricky. For obesity, evidence is preliminary; while some rodent studies are promising, human evidence that fermented-food peptides cause weight loss is almost nonexistent at this point. On the other hand, given the global interest in functional foods, proponents are excited by any positive signal. They argue that incorporating peptide-rich fermented foods into the diet could have cumulative metabolic benefits.

In conclusion, peptides from fermented foods show potential cardiometabolic protective effects across multiple fronts: lowering blood pressure, assisting glycemic control, improving lipid profiles, and modulating oxidative and inflammatory stress. The strength of evidence differs substantially by outcome. Blood-pressure evidence is the most clinically developed but modest and heterogeneous; glycemic and inflammatory evidence is mechanistically plausible but less directly proven in humans; lipid and weight evidence is still early. These peptides are therefore best viewed as contributors to the health potential of fermented foods rather than as stand-alone therapeutics.

## Challenges, controversies, and future perspectives

Despite the encouraging findings reviewed above, significant challenges and controversies remain in harnessing bioactive peptides from fermented foods for cardiometabolic health. These challenges span scientific, technological, and regulatory domains. Here we discuss the major issues: variability in peptide production and potency, bioavailability and stability concerns, the complexity of proving efficacy in humans, and regulatory hurdles. We also outline future research directions and innovations that may address these challenges, aiming to move the field from promising research to practical health applications.

Variability in Peptide Content and Reproducibility: One fundamental challenge is that the content and profile of bioactive peptides in fermented foods can be highly variable. Fermentation is a complex biological process sensitive to strains, substrate, and conditions. A yogurt made with one *Lactobacillus* strain may contain a very different set of peptides than another yogurt with a different culture. Even the same fermented product can vary batch-to-batch. For example, the concentration of IPP and VPP in commercial fermented milk products was found to range widely depending on manufacturing processes. This variability complicates research and consumer use. Standardization is difficult because one must control the microbial enzymes’ activity. Industrial solutions being explored include using starter cultures that are highly proteolytic in a consistent way or adding specific proteases to drive production of target peptides. For instance, isolating or engineering a *Lactobacillus* strain that overexpresses a particular proteinase can yield more of a desired antihypertensive peptide consistently. Some companies have patented fermentation processes to maximize IPP/VPP output in milk. Nonetheless, achieving uniform peptide levels in traditional fermented foods is inherently challenging.

Bioavailability and Stability in the Gut: A critical question is how well these peptides survive digestion and reach their sites of action. The gastrointestinal tract is a gauntlet of proteases that will cleave many peptides into amino acids or very short fragments. Some peptides, especially Pro-containing sequences such as IPP and VPP, are relatively resistant and can be absorbed intact [61]. In contrast, many longer or highly charged peptides may act mainly in the gut lumen, for example by binding ACE locally, interacting with bile acids, modulating microbes, or neutralizing luminal LPS. This distinction explains why a peptide can show excellent IC50 values in vitro but limited systemic activity after ingestion. Encapsulation, matrix protection, and peptide-enriched fermentation are being explored to improve delivery [62–63].

Efficacy in Humans: Designing and interpreting human trials for functional foods is inherently tricky. Diet is complex, and placebo control is challenging. Many early studies showing large benefits may have overestimated effects due to placebo or publication bias. Later, more rigorous trials often found smaller or no effects, fueling controversy. For example, early open-label trials of fermented milk in hypertensives reported strong BP reductions, but when replicated in double-blind fashion, the effect shrank or vanished. Some have raised the possibility that uncontrolled lifestyle factors could contribute to observed improvements in metabolic parameters, rather than the peptides per se. To convince skeptics, large well-controlled trials are needed. However, these are expensive and logistically hard for a food (which cannot be patented like a drug). This leads to a catch-22: without clear evidence, regulatory agencies won’t approve health claims, and without proprietary protection, companies are reluctant to fund large studies. So the field is somewhat stuck with many small to medium trials of varying quality. This has perpetuated debate, as one can cherry-pick positive or negative studies to support either side. A way forward is through independent meta-analyses and consensus statements. For instance, the International Dairy Federation and other expert groups have reviewed the lactotripeptide literature and generally conclude a mild BP-lowering effect exists, while urging further research. Yet, authoritative bodies like EFSA remain unconvinced. The difference in global regulatory acceptance is noteworthy: Japan and some Asian countries allow BP-lowering claims on lactotripeptide products, whereas the EU and US do not. This regulatory controversy influences the market and research funding environment.

Safety and Side Effects: On the whole, fermented food peptides are considered safe. No serious adverse effects have been attributed to them in trials. Unlike pharmaceuticals, they are broken down to amino acids eventually and do not accumulate. However, one theoretical concern is allergenic potential: some peptides might be epitopes for food allergies. Another is that ACE-inhibitory peptides, if extremely potent, might cause hypotension or interact with medication – but given their modest effect, this is unlikely. Studies have not reported excessive BP lowering or any ACE-inhibitor-like cough with these peptides. DPP-IV inhibitory peptides raise a different question: DPP-IV is involved in peripheral metabolism of many peptides. Pharmaceutical DPP-IV inhibitors (gliptins) have been associated with a slight risk of infections and pancreatitis in some cases. But the much weaker inhibition by food peptides likely poses minimal risk. Nonetheless, long-term consumption of potent DPP-IV inhibitors from diet hasn’t been deeply studied; could it impair immune surveillance? Current evidence suggests not, given how mild the inhibition is. Overall, safety is a lesser concern but needs to be monitored as these peptides move toward functional “nutraceutical” use in concentrated forms.

The Complexity of Food Matrices and Synergy: A recurring theme is that in a whole food, peptides act in concert with other nutrients. This complicates attribution but also offers opportunity for synergistic effects. For example, the combination of lactotripeptides and plant sterols in a functional milk drink improved lipids and possibly BP. Fermented foods naturally provide such combinations: kefir has peptides, probiotics, and polysaccharides; tempeh has peptides along with soy isoflavones; kimchi has peptides from fish sauce plus vitamins from vegetables. These synergistic mixtures might be more effective at improving cardiometabolic health holistically than an isolated peptide supplement. The flip side is that isolating the cause of benefits becomes challenging. Going forward, embracing a systems approach might be better: rather than crediting a single peptide, one can promote a fermented food as a whole for health, recognizing that peptides are one of several beneficial components. This is indeed how traditional diets worked – e.g, diets including yogurt have lower incidence of metabolic syndrome, not because of one peptide but due to multiple factors in yogurt. But for the science of structure–function, teasing out the peptide’s role still holds value, as it enables optimizing those factors.

Regulatory and Public Acceptance: Regulatory bodies differ in their assessment of peptide-related health claims. In Europe, EFSA has required convincing human evidence that a defined peptide preparation causes a beneficial physiological effect, and the IPP/VPP blood-pressure claim was not substantiated [46]. In other regions, fermented milk products containing IPP/VPP have been marketed with stronger functional-food positioning. These regulatory disparities are best understood as differences in evidentiary thresholds rather than simple disagreement about mechanism. Future submissions will be stronger if they combine standardized peptide quantification, matched fermented and non-fermented controls, clinically meaningful endpoints, and transparent reporting of null as well as positive findings.

Future Perspectives: The field is moving toward greater precision. Important directions include strain selection to enrich reproducible peptide profiles, peptidomic monitoring of fermented foods, in silico screening to identify multi-target sequences, formulation strategies that protect peptides during digestion, and clinical trials that pair peptide exposure biomarkers with cardiometabolic endpoints. The most useful next generation of studies will compare whole fermented foods, matched non-fermented controls, and defined peptide fractions within the same design, thereby clarifying how much of a food’s effect can reasonably be assigned to peptides.

- Bioengineering of strains to yield higher peptide titers or even novel peptides. For instance, using recombinant LAB that express fungal proteases to generate unique peptide profiles not found in natural fermentations. Synthetic biology could create a *Lactococcus* that consistently churns out a tripeptide of choice in a fermenter, enabling production of peptide-enriched fermented ingredients.
· Peptide libraries and in silico screening: Researchers have compiled databases of food-derived and fermented-food peptides listing sequences and activities. Machine learning on these datasets may predict new peptide sequences with multi-target benefits. These candidates can then be produced by microbial fermentation, enzymatic hydrolysis, or synthetic routes for testing. Computational predictions should be treated as prioritization tools, not substitutes for digestion, bioavailability, and human-outcome validation.
· Combination products: as mentioned, combining peptides with other functional compounds may produce additive or synergistic effects. Already, some commercial products pair peptides with minerals like magnesium or with probiotics. The concept of “synbiotics” can extend to “syn-nutraceuticals” where a peptide and a probiotic and a fiber might collectively improve a cluster of syndrome features.
· Clinical targeting: Instead of broad public use, fermented food peptides might find a niche in specific clinical scenarios – e.g, managing prehypertension, or gestational diabetes, or in frail elderly. Tailoring functional foods to such groups could maximize risk-benefit.

Addressing controversies with robust research: The controversies can be resolved with well-designed trials – e.g. a large multicenter trial in both Europe and Asia using the same peptide product to see if ethnicity truly matters, or long-term metabolic outcome studies. Such studies are expensive and require collaboration between academia, industry, and possibly government health bodies who have interest in preventive approaches. If conducted, they could provide definitive answers and either silence skepticism or redefine how we view these nutraceuticals.

In conclusion, bioactive peptides from probiotic fermented foods lie at the exciting intersection of nutrition and pharmacology. They offer a natural means to modulate key pathways implicated in cardiometabolic diseases. While evidence for their benefits is substantial, it is not yet uniform or universally accepted, leading to ongoing debate. Challenges in production, standardization, and demonstration of efficacy need to be met with interdisciplinary efforts. The future likely holds more targeted uses of these peptides. With rising rates of metabolic syndrome globally, even small contributions from functional foods can translate to large public health gains. Continued research and open scientific dialogue will be critical to fully realize and appropriately harness the cardiometabolic protective potential of fermented food–derived bioactive peptides.

## Conclusion

Fermentation has long been used to enhance foods, but only in recent decades we have begun to unravel how fermentation endows foods with specific health-promoting molecules. Bioactive peptides generated by probiotic microbes are emerging as key nutraceutical components that may contribute to the observed benefits of fermented diets on cardiometabolic health. This critical review has examined the evidence that such peptides can improve blood pressure, modulate glucose homeostasis, improve lipid profiles, and reduce inflammation. The bioactive peptides from fermented foods show multifaceted cardiometabolic potential, with the strongest human evidence for modest blood-pressure reduction and more preliminary evidence for incretin-mediated glycemic effects, lipid modulation, and inflammatory control. Peptides such as IPP and VPP act as natural ACE-inhibitory sequences and have lowered blood pressure in some, but not all, human trials. Fermentation-derived peptides can also inhibit DPP-IV, scavenge radicals, bind LPS, or influence lipid-related pathways in experimental models. The important qualification is that mechanistic potency does not guarantee clinically meaningful effects after digestion and food-matrix dilution.

However, the translation of these findings to consistent human health benefits faces challenges and has sparked debate. Core arguments challenging the impact of fermented food peptides include the variability and typically small magnitude of effects in human studies, especially in diverse populations. Detractors note that while certain trials in Japan showed ~5–8 mmHg blood pressure reductions, others in Europe showed virtually no effect. They question whether factors like genetic background, diet, or even placebo effect might explain positive findings more than the peptides themselves. Similarly, the glycemic benefits, though mechanistically sound, have mostly been demonstrated acutely or in animal studies – consistent long-term improvements in human diabetes outcomes remain to be proven. Skeptics also emphasize that fermented foods contain many active components, so isolating the contribution of peptides is complex; some argue that any health benefit could be due to these other factors rather than the peptides. Indeed, the controversy often centers on causation and attribution: are peptides truly the “active drug” within fermented foods, or are they one of many contributors to a generally healthy food?

On the supporting side, proponents cite the substantial body of evidence from controlled experiments demonstrating peptide-specific activities. They point out that multiple peer-reviewed studies have identified specific peptide sequences from foods and shown their ability to modulate key physiological targets in ways that align with improved cardiometabolic health. For example, scholars in favor highlight that the International Union of Nutritional Sciences has recognized lactotripeptides as having a genuine antihypertensive effect based on aggregate data. They also note instances where peptides were isolated and given in pill form and still produced the expected effect, reinforcing that the peptide itself was efficacious. Proponents argue that the focus of controversy should shift from “do they work at all” to “how can we enhance and reliably reproduce their benefits,” given that the mechanistic plausibility and a sizeable fraction of trials support efficacy.

The focus of the controversy thus lies in the consistency, generalizability, and practical significance of the peptide effects. Both sides agree that these peptides are biologically active; the debate is over whether they can reliably improve health outcomes in free-living humans enough to matter clinically. Issues such as optimal dosage, inter-individual differences, and ensuring stability/bioavailability are at the forefront of discussion. To address these, future research is honing in on precision nutrition approaches – identifying which subgroups respond best to peptide intake, and how to formulate peptide-rich foods for maximal efficacy.

In moving forward, there is broad agreement that fermented foods can be valuable components of healthy dietary patterns. Bioactive peptides likely contribute to this value in some foods and populations, alongside probiotics, minerals, organic acids, vitamins, fiber, and other matrix components. A balanced interpretation is that fermented-food peptides may help tune cardiometabolic pathways over time, especially when consumed as part of a broader lifestyle strategy, but the field should avoid overstating peptide-specific efficacy until trials quantify peptide exposure and clinically relevant endpoints together.

In conclusion, bioactive peptides from probiotic fermented foods represent a promising, if nuanced, tool in the nutritional prevention and management of cardiometabolic diseases. They exemplify the concept of food as medicine, where ancient dietary practices dovetail with modern biochemical insights. Ongoing research and well-designed clinical studies will be critical to fully establish their role and to convince all stakeholders of their benefits. If current positive trends are confirmed, we can envision a future where fermented functional foods enriched in specific peptides are recommended alongside other interventions to help curb the global tide of cardiometabolic disorders. As with any dietary strategy, they are not a standalone cure-all, but as part of an integrated approach, these natural peptides offer a safe and accessible means to support cardiovascular and metabolic health. The journey from fermented food to functional peptide and from bench to bedside is still unfolding, but it holds much potential for innovation in public health nutrition and preventive cardiology.

## Figures and Tables

**Table 1:** Representative bioactive peptides identified in probiotic fermented foods.

Fermented food	Representative peptide(s)	Main reported activity	Evidence note
*L. helveticus fermented milk*	IPP and VPP	ACE inhibition; modest blood-pressure lowering	Classic lactotripeptides; human effects vary by population and trial design [1,55].
*Cheddar cheese*	VLPVPQK	Antioxidant activity	Lactobacillus fermentation can release radical-scavenging casein fragments [64].
*Fermented beef*	IVG	Antihypertensive activity	A fermented beef peptide lowered blood pressure in SHR models [4].
*Natto*	KFNKYGR; FPFPRPPHQK	LPS neutralization; angiogenic activity	Cationic natto peptides support anti-inflammatory mechanistic plausibility [10].
*Fermented tomato waste proteins*	DGVVYY	ACE inhibition	Bacillus fermentation generated ACE-inhibitory peptides including DGVVYY [18].
*Fermented cheese/rubing*	Multiple short peptides	ACE, antioxidant, alpha-glucosidase inhibition	Peptidomics links fermented dairy peptides with multi-target activity [6].
*Fermented fish or shrimp paste*	Hydrophobic peptide fractions	ACE inhibition; antioxidant activity	Traditional fermented marine products provide peptide-rich matrices [7,65].
*Fermented broad bean paste*	LY-4, LP-5, VL-9	Nrf2-linked antioxidant protection	Recent in vitro work supports strain- and substrate-specific peptide profiles [26].
*Rice bran fermentation*	Alpha-glucosidase inhibitory oligopeptide	Antidiabetic enzyme inhibition	Plant fermentation can generate peptides relevant to postprandial glucose handling [66].

**Table 2:** Structural features of bioactive peptides and their associated activities.

Activity	Typical structural features	Examples and interpretation
*ACE inhibition*	Short peptides; hydrophobic/aromatic C-terminal residues; frequent Pro	IPP/VPP and DGVVYY illustrate Pro- or Tyr-rich ACE-inhibitory motifs [16–17].
*DPP-IV inhibition*	Di- to pentapeptides; N-terminal Pro/Ala or bulky hydrophobic residues	Food-protein peptides can inhibit DPP-IV in vitro, but human exposure remains uncertain [29,52].
*Antioxidant activity*	Hydrophobic, aromatic, sulfur-containing, or His-rich residues	Aromatic and sulfur-containing residues support radical scavenging and metal chelation [20,67].
*Anti-inflammatory activity*	Cationic Arg/Lys-rich or amphipathic sequences; selected Pro-Gly-Pro motifs	Natto cationic peptides neutralize LPS in vitro; clinical biomarker evidence is limited [10,36].
*Lipid/adiposity pathways*	Hydrophobic peptides that bind bile acids or influence AMPK/PPAR pathways	Mechanistic evidence is plausible, but food-matrix attribution remains difficult [56,58].
*Bioavailability*	Protease resistance, small size, Pro-rich motifs, or matrix protection	Absorption and stability determine whether in vitro potency can translate *in vivo* [63,61].

**Table 3:** Selected evidence on antihypertensive effects of fermented food-derived peptides.

Study/model	Intervention	Blood-pressure outcome	Interpretation
*Hata et al., Japan RCT*	Sour milk with IPP/VPP	Marked SBP/DBP reduction	Early positive proof of concept in hypertensive adults [43].
*Engberink et al., Netherlands RCT*	IPP/VPP-enriched yogurt drink	No clinically relevant 24-h BP reduction	High-quality European trial showing null effect [45].
*EFSA opinion*	Review of IPP/VPP claim	Causality not substantiated	Concern centered on inconsistent human evidence, not absence of ACE mechanism [46].
*Qin et al., meta-analysis*	Lactotripeptide RCTs	Small pooled BP reductions	Mean effects were statistically significant but modest and heterogeneous [47].
*Boelsma and Kloek RCT*	IPP-rich milk-protein hydrolysate	Office BP lowered in stage 1 hypertension	Defined hydrolysate evidence should be separated from whole-food evidence [55].
*Recent peptide meta-analysis*	Food-protein-derived peptides	Overall BP-lowering signal	Supports activity of peptide preparations but includes heterogeneous sources [68].
*SHR and animal models*	Purified or enriched peptides	Often larger BP reductions than human trials	Animal potency confirms mechanism but can overestimate clinical effect [4,41].
